# Synchronous Macular Amyloidosis and Brachioradialis Pruritus Successfully Treated With Gabapentin

**DOI:** 10.1155/crdm/5909428

**Published:** 2025-11-26

**Authors:** Hoda Rahimi, Mehrdad Ashayer, Roya Zeinali, Leila Rezaie Shirmard

**Affiliations:** ^1^Skin Research Center, Shahid Beheshti University of Medical Sciences, Tehran, Iran; ^2^Department of Dermatology, Rasool Akram Medical Complex Clinical Research Development Center (RCRDC), School of Medicine, Iran University of Medical Sciences (IUMS), Tehran, Iran; ^3^Department of Pharmaceutics, School of Pharmacy, Ardabil University of Medical Sciences, Ardabil, Iran

## Abstract

Brachioradial pruritus (BRP) and macular amyloidosis (MA) are distinct dermatological conditions that have rarely been reported together. BRP is a neuropathic pruritus affecting the lateral forearm, whereas MA presents as hyperpigmented patches caused by amyloid deposition in the dermis. We report the first case of synchronous MA and BRP in a 45-year-old woman who presented with severe pruritus in the brachioradialis distribution, concurrent with hyperpigmented patches characteristic of MA. Cervical spine imaging revealed moderate-to-severe multilevel degenerative changes. The patient was successfully treated with gabapentin 200 mg daily, with significant improvement in both conditions. This case highlights the first reported synchronous presentation of MA and BRP and suggests potential shared pathophysiological mechanisms. The favorable dual therapeutic response to gabapentin provides new insights into management strategies for concurrent presentations of these conditions.

## 1. Introduction

Brachioradial pruritus (BRP) is a neuropathic pruritus characterized by intense itching, burning, or stinging sensations along the lateral forearm in the distribution of the brachioradialis muscle, without primary cutaneous lesions [1, 2]. It is believed to result from cervical nerve root compression or irritation, typically at the C5–C8 levels, and predominantly affects middle-aged individuals with a history of sun exposure [3, 4].

Macular amyloidosis (MA) is a form of primary localized cutaneous amyloidosis characterized by hyperpigmented, rippled patches, most commonly on the upper back, chest, and arms. It arises from the deposition of keratinocyte-derived amyloid fibrils in the papillary dermis, leading to its characteristic clinical and histopathological features [5].

Although both conditions can affect the upper extremities and are associated with pruritus, their synchronous presentation has not been previously reported in the medical literature. The concurrent occurrence of these seemingly distinct conditions raises important questions about shared pathophysiological mechanisms and therapeutic approaches.

Both conditions are chronic, with pruritus significantly impairing quality of life [1, 2]. No treatment strategies have been proven to treat MA, and the treatment emphasizes breaking itch–scratch–itch cycle, including topical corticosteroids in mild cases, chemical peels, phototherapy, and lasers. Tables 1 and 2 outline treatment options for BP and MA, respectively. Recent studies suggest that gabapentin is effective in managing BRP [6–8]. Gabapentin, a γ-aminobutyric acid (GABA) analogue, inhibits voltage-dependent calcium channels, thereby suppressing the sensation of itch [9].

Here, we report the first case of BRP associated with MA in a patient successfully managed with low-dose gabapentin.

## 2. Case Presentation

A 45-year-old woman with no significant past medical history presented to our dermatology clinic with a 6-month history of severe pruritus and a burning sensation in her left arm. Her medical history included ischemic heart disease, hypertension, and knee osteoarthritis, all controlled with medication. She reported no family history of neurological or dermatological disorders, no recent travel, and no relevant environmental exposures.

The severity of her pruritus was measured using a visual analog scale (VAS) and rated 8 points out of 10. Symptoms markedly impaired her quality of life, disrupting sleep, and daily activities. She had used topical steroids and emollients for 3 months without improvement.

**Figure 1:**
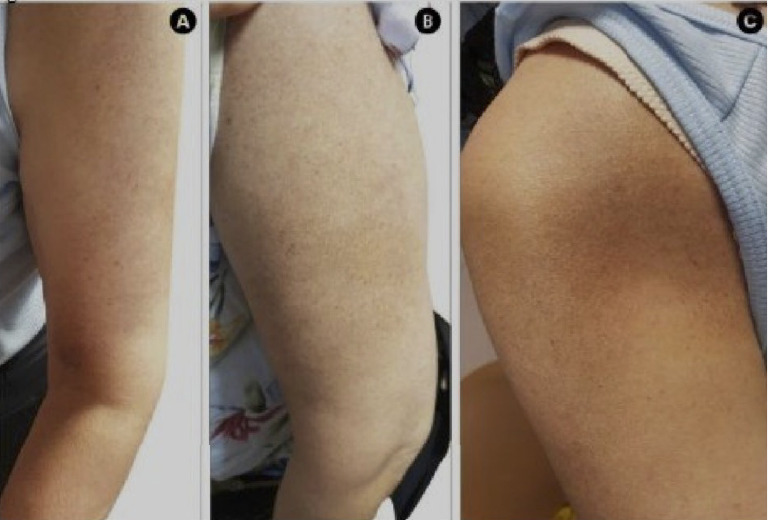
Skin involvement in a patient with BRP. No solar damage or specific skin lesions were observed, except for macular amyloidosis on her left arm.

On examination, no solar damage was observed. Hyperpigmented patches with a rippled appearance, consistent with MA, were present on the left arm (Figure 1). Neurological examination revealed hyperesthesia in the brachioradialis distribution, with preserved motor function and reflexes. Although the patient denied neck pain, discomfort was elicited upon neck extension.

**Figure 2:**
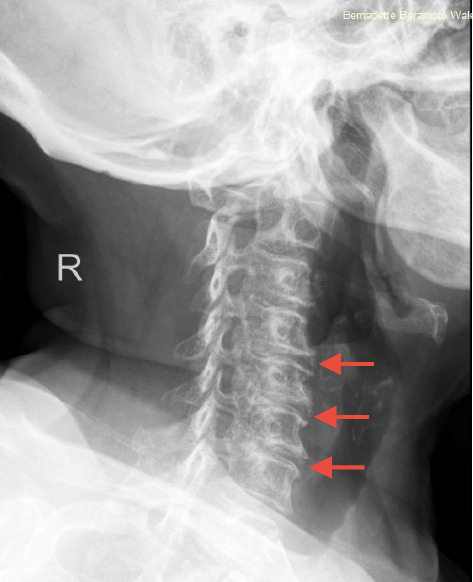
A cervical spine X-ray of the patient reveals moderate-to-severe multilevel degenerative changes in the C4-C5, C5-C6, and C6-C7 intervertebral regions (arrows).

A cervical spine X-ray was performed, revealing moderate-to-severe multilevel degenerative changes in the C4-C5, C5-C6, and C6-C7 intervertebral regions (Figure 2).

Based on the physical examination, the patient was diagnosed with synchronous MA and BRP. She was prescribed gabapentin 100 mg twice a day and triamcinolone ointment twice a day and advised on measures to reduce pressure on the cervical vertebrae. At 2-month follow-up, pruritus intensity had decreased to 3/10 on the VAS, with marked improvement in sleep and daily function. Additionally, the MA lesions appeared lighter. No adverse effects were reported.

## 3. Discussion

This case describes a 45-year-old woman with synchronous MA and BRP who responded favorably to gabapentin. BRP is considered a chronic sensory neuropathy, most commonly linked to cervical spine pathology at the C5–C7 levels, which may present with pruritic dermatoses of the arms and hands [4]. Typically, affected skin lacks primary lesions aside from excoriations or prurigo due to scratching. Hence, neurological examination and cervical imaging are crucial when BRP is suspected [8]. In our patient, despite the absence of sun damage (likely related to cultural clothing practices), cervical pathology was demonstrated radiographically. The use of topical agents prior to presentation may explain the minimal secondary lesions.

This is the first reported case of synchronous MA and BRP, with several important clinical implications. Notalgia paresthetica, another neuropathic itch disorder, is frequently associated with MA [10]. The pathogenesis of MA remains unclear, but chronic mechanical trauma is strongly implicated. Persistent scratching or friction is thought to trigger keratinocyte apoptosis and subsequent amyloid deposition in the papillary dermis. In this case, chronic neuropathic itch from BRP in the C5-C6 dermatomes likely contributed to amyloid formation through repetitive mechanical stress.

Gabapentin, a GABA analogue, exerts antipruritic effects by binding the *α*2*δ* subunit of voltage-gated calcium channels, thereby reducing excitatory neurotransmitter release from sensory neurons. Its neuromodulatory properties make it effective in several neuropathic itch syndromes. In BRP, gabapentin has been consistently reported as effective when conventional therapies fail [6–8, 11].

Although gabapentin's role in MA is less established, its efficacy in this case suggests a neuropathic component to MA-associated pruritus [10]. By reducing abnormal neuronal signaling, gabapentin may relieve both BRP-associated itch and interrupt the itch–scratch cycle in MA. Indeed, a recent randomized trial demonstrated significant pruritus relief with topical gabapentin in primary MA, supporting its role in amyloid-associated itch [12]. In practice, treating both conditions with a single agent reduces polypharmacy and improves compliance, and it supports the idea of a shared pathophysiology that gabapentin addresses.

Our patient experienced marked improvement at a relatively low dose (200 mg/day) within one week. Other reports describe effective doses ranging from 900 to 3900 mg/day [2, 6, 8, 13]. We therefore recommend starting with a low dose and titrating upward based on response and tolerability.

Gabapentin has a favorable safety profile, particularly compared to other antipruritic therapies [14]. It is well tolerated in elderly patients and those receiving multiple medications, where drug interactions are a concern.

## 4. Conclusion

BRP may coexist with MA, and both conditions can be effectively managed with gabapentin. Initiating therapy at a low dose and adjusting according to clinical response is recommended. Further large-scale studies are warranted to clarify the association between BRP and MA, establish optimal gabapentin dosing protocols, and compare its efficacy with other treatments.

## Figures and Tables

**Table 1:** Common treatment options for brachioradialis pruritus.

Treatment modality	Mechanism of action	Efficacy	Adverse effects/limitations	Level of evidence
*Topical*				
Capsaicin (topical cream, 0.025%–0.1% [15]; 8% patch [16])	TRPV1 agonist causing C-fiber defunctionalization	77% patients reported “itch much improved or gone” after 3 weeks of 0.025%–0.1% cream	Acute burning/stinging during application, erythema	Case series
		Significant itch and paresthesia reduction (*p* < 0.05) in a study of 31 patients.		
Compounded topical ketamine–amitriptyline [17]	Ketamine: NMDA receptor antagonist; amitriptyline: TCA (blocks reuptake of serotonin/norepinephrine)	Relieve itch in refractory cases	Local skin irritation; potential systemic absorption	Case series
Topical anesthetics	Sodium channel blockade	Temporary relief, variable response	Contact dermatitis, sensitization	Expert opinion

*Systemic*				
Gabapentin [6, 7, 13]	GABA analogue; binds the *α*2*δ* subunit of voltage-gated Ca2+ channels, reducing excitatory neurotransmitter release	70%–90% response rate, especially at higher doses (e.g., ≥ 1,500 mg/day)	Dizziness, sedation, peripheral edema, ataxia, weight gain	Case series
		VAS reduction 3–5 points		
Pregabalin [18]	Similar to gabapentin (*α*2*δ* Ca2+ subunit binding); neuromodulatory.	Similar to gabapentin, 60%–80% response	Drowsiness, dizziness, weight gain (reports of up to 15%–20% pts)	Case series
Aprepitant [19]	Blocks neurokinin-1 receptor, preventing substance P–mediated pruritus signaling	Some improvement in refractory BRP in a 6-week case report	Well -tolerated; some patients report fatigue and nausea	Case report
Dupilumab [20]	IL-4/IL-13 pathway blockade	95% pruritus improvement of recalcitrant case within the first 3 months	N/A	Case report
Naloxone (IV) [21]	μ-Opioid receptor antagonism	Marked itch reduction, WINRS decreased from 8–10/10 to 2–4/10%; 70%–90% improvement in dynamic pruritus; sustained benefit at 6 months in 2/4 patients	No adverse events	Case series
Doxepin	Antihistaminic, TRP modulation	Limited documented efficacy	Variable tolerability	Expert opinion
Antihistamines	H1 receptor blockade	Generally ineffective, not effective for neuropathic itch	N/A	Expert opinion

*Procedure*				
Botulinum toxin A [22]	Inhibition of acetylcholine release; modulation of sensory signaling	Dramatic relief lasting 5–6 months	Limited controlled data; injection discomfort; speculative mechanism	Case report
Cervical epidural steroid injection (CESI) [23]	Anti-inflammatory steroid delivery to reduce nerve root inflammation	71-year-old with stenosis: Marked improvement after 2 CESIs at C6-C7	Effects may be temporary	Case report
Anterior cervical discectomy and fusion (ACDF)	Definitive decompression of pathological cervical discs/foramina	Complete resolution after ACDF; one required revision after cage migration	Surgical risks; recurrence if hardware issues; applicable mainly to compressive pathology	Case report
Chiropractic care [24]	Spinal manipulation to reduce mechanical cervical nerve irritation	Symptom resolution in single case	Anecdotal; not reproducible in controlled fashion	Case report

**Table 2:** Common treatment options for macular amyloidosis.

Treatment modality	Mechanism of action	Efficacy	Adverse effects/limitations	Level of evidence
*Topical*				
High-potency corticosteroids	Anti-inflammatory; reduce itch and local epidermal drive to amyloid formation	Often minimal improvement in pigmentation; can modestly reduce pruritus; small series suggest transient relief	Skin atrophy, telangiectasia with chronic use	Expert opinion
			Limited standalone pigment clearance; best used adjunctively	
Topical retinoids (tazarotene, tretinoin) [25]	Normalize keratinocyte differentiation; accelerate epidermal turnover	Gradual reduction of pigmentation and scaling over months; in combination (e.g., with CO_2_ laser), all patients improved	Irritation, peeling, photosensitivity	Case series
Tacrolimus [26]	Inhibits T-cell-mediated inflammation in skin	Itch reduction and some pigment fading	Local burning or irritation	Case report/expert opinion
Gabapentin (6% cream) [12]	Binds neuronal *α*2*δ* Ca2+ subunit; neuromodulator	Significantly reduced pruritus scores and produced short-term pigment lightening	None	RCT
Capsaicin	Activates TRPV1, depleting substance P in skin nerve endings	Pruritus improvement; pigment effects unclear	Burning sensation at application	Expert opinion

*Systemic*				
Colchicine [27]	Anti-inflammatory and anti-fibrotic effects; may reduce amyloid deposition and pruritus	100% pruritus resolution in MA patients; pigmentation almost disappeared within 90 days	None	Case series
Acitretin [28]	Normalize keratinocyte differentiation; reduce amyloid precursor formation	Successful improvement in a patient with severe biphasic cutaneous amyloidosis	Dry skin, mucosal dryness, teratogenicity	Case report
Oral antihistamines or TCAs (e.g., amitriptyline)	Sedating antihistamine or centrally acting TCA; may break itch–scratch cycle	Variable and generally limited effect on pigment; may modestly reduce pruritus in some patients	Sedation, anticholinergic (dry mouth, etc.) for TCAs	Expert opinion

*Procedure*				
Fractional CO_2_ laser [29]	Ablative fractional photothermolysis induces transepidermal elimination of amyloid and dermal remodeling	Superficial mode: greater pigmentation reduction with better tolerability; follow-up 3 months to 20 weeks with low recurrence short term		
The most-effective laser treatment of MA	Pain (higher with deep mode), transient erythema	RCT		
Fractional Er:YAG laser [30]	Ablative fractional resurfacing with dermal remodeling	Significant clinical and histological improvement at 3-month follow-up	Generally, well tolerated	Case series
Q-switched Nd:YAG laser [31]	Target melanin and pigment; selective photothermolysis	532 nm more effective than 1,064 nm (90% vs. 60% good/very good); ∼36.7% achieved > 50% improvement; ∼66% satisfaction	Mild transient side effects	Cohort studies
Fractional nonablative erbium 1540 nm [32]	Nonablative fractional heating to induce dermal remodeling	Suitable and efficacious without major complications, less downtime than ablative lasers	Mild transient side effects	RCT
PDL (pulsed dye laser) [33]	Vascular targeting	Variable efficacy; less effective than CO_2_	N/A	Comparative reports
Chemical peel (TCA, glycolic acid) [34]	Chemical exfoliation	In a head-to-head RCT: 15% trichloroacetic acid peel produced superior pigment reduction versus 35% glycolic acid peel and QS Nd:YAG laser; glycolic acid gave moderate improvement	Erythema, irritation; risk of postinflammatory hyperpigmentation if mishandled	RCT
Ultraviolet B phototherapy [35]	UV-induced immunomodulation + anti-inflammatory	Successful management of pruritus	Burning, photoaging, carcinogenesis	Case report
Microneedling [36]	Dermal remodeling + melanogenesis inhibition	Pruritus improved ∼61.7% after four sessions; similar efficacy with or without tranexamic acid	Discomfort	RCT

## Data Availability

The data that support the findings of this study are available from the corresponding author upon reasonable request.
